# Estimation of acute and chronic Q fever incidence in children during a three-year outbreak in the Netherlands and a comparison with international literature

**DOI:** 10.1186/s13104-015-1389-0

**Published:** 2015-09-18

**Authors:** Edwin N. E. Slok, Frederika Dijkstra, Esther de Vries, Ariene Rietveld, Albert Wong, Daan W. Notermans, Jim E. van Steenbergen

**Affiliations:** National Institute for Public Health and the Environment, Centre for Infectious Disease Control, P.O. Box 1, 3720 BA Bilthoven, The Netherlands; Department of Paediatrics, Jeroen Bosch Hospital, ‘s-Hertogenbosch, The Netherlands; Department of Infectious Disease Control, Municipal Health Service ‘Hart voor Brabant’, ‘s-Hertogenbosch, The Netherlands; Department of Statistics, Mathematical Modelling and Data Logistics, National Institute for Public Health and the Environment, P.O. Box 1, 3720 BA Bilthoven, The Netherlands; Centre for Infectious Diseases, Leiden University Medical Centre, P.O. Box 9600, 2300 RC Leiden, The Netherlands

**Keywords:** Acute, Chronic, Q fever, Children, Outbreak, Epidemic, Seroprevalences studies

## Abstract

**Background:**

In the Dutch 2007–2009 Q fever outbreak *Coxiella burnetii* was transmitted aerogenically from dairy goat farms to those living in the surrounding areas. Relatively few children were reported. The true number of pediatric infections is unknown. In this study, we estimate the expected number of acute and chronic childhood infections.

**Methods:**

As Coxiella was transmitted aerogenic to those living near infected dairy goat farms, we could use adult seroprevalence data to estimate infection risk for inhabitants, children and adults alike. Using Statistics Netherlands data we estimated the number of children at (high) risk for developing chronic Q fever. Literature was reviewed for childhood (0–15 years) Q fever reports and disease rates. We compared this with Dutch reported and our estimated data for 2007–2009.

**Results:**

In The Netherlands epidemic, 44 children were reported (1.2 % of total notifications). The childhood incidence was 0.15 compared to 2.6 per 10,000 inhabitants for adults. No complications were reported. Based on the expected similarity in childhood and adult exposure we assume that 9.8 % of children in the high-risk area had Q fever infection, resulting in 1562 acute infections during the Q fever epidemic interval. Based on the prevalence of congenital heart disease, at least 13 children are at high risk for developing chronic Q fever. In medical literature, 42 case reports described 140 childhood Q fever cases with a serious outcome (four deaths). In chronic Q fever, cardiac infections were predominant. Four outbreaks were reported involving children, describing 11 childhood cases. 36 National and/or regional studies reported seroprevalences varying between 0 and 70 %.

**Conclusion:**

In the 3-year Dutch epidemic, few childhood cases were reported, with pulmonary symptoms leading, and none with a serious presentation. With an estimated 13 high-risk children for chronic infection in the high exposure area, and probably forty in the whole country, we may expect several chronic Q fever complications in the coming years in paediatric practice.

**Electronic supplementary material:**

The online version of this article (doi:10.1186/s13104-015-1389-0) contains supplementary material, which is available to authorized users.

## Background

Between 2007 and 2009, a three-year cumulative epidemic of Q fever occurred, mainly in the Southern part of the Netherlands [1]. Over this period, more than 4000 patients were reported to Municipal Health Services (MHS).

The most important mode of transmission during this epidemic was an airborne spread of the causative micro-organism *Coxiella burnetii* from infected dairy goat farms to surrounding living areas. Exposure risk was related to proximity of living near infected farms for adults and children [2–5]. The epidemic ended after vaccination of the complete Dutch dairy goat population.

During this epidemic hardly any children under the age of 15 years were reported [6]. This could be expected as Q fever in children has been described as mild or asymptomatic (60 %), only sporadically leading to serious complications [2, 7–10]. The most reported “classical” symptom is unexplained fever, a query, attributing to its name [11].

Many mild *Coxiella* infections may have been missed in the Netherlands because general practitioners generally do not perform laboratory tests in children with self-limiting fever of unknown origin. The Dutch guideline “Children with fever” (M29, May 2008) of the Dutch College of General Practitioners (NHG) recommends to perform only urinalysis for leukocytes and nitrite in mildly ill children, and to consider performing a chest X-ray in case of suspected pneumonia. Microbiological diagnostic tests will rarely be performed in mildly ill children with fever. Therefore, the true number of Q fever in children during the Dutch epidemic remains unknown. Furthermore, all asymptomatic infections of course remained undetected.

*Coxiella* infected individuals with particular conditions, e.g. immunosuppression, congenital heart disease, heart valve lesion or vascular abnormalities are more likely to develop chronic Q fever [12–14]. Thus, in the coming years an unknown number of chronic Q fever may become unveiled in the geographical area where Q fever cases were abundant.

In this article, we present an overview of reported Q fever cases during the 2007–2009 epidemic in The Netherlands. Based on this, we have estimated the likely total number of childhood infections, and subsequently the expected number of chronic infections in high-risk children, and we compare this with reports in medical literature.

## Methods

### Dutch reports

In the Netherlands, physicians are statutorily required to report Q fever patients to the Municipal Health Service (MHS). All Dutch laboratories report positive serological findings of recent Q fever to the MHS. The MHS takes local action and daily reports anonymised patient data through the electronic registration system of legally reported infectious diseases (Osiris) to the Centre of Infectious Diseases Control of the National Institute for Public Health and the Environment (RIVM). Amongst others, age, gender and postal code are registered. Reporting criteria for Q fever are fever, or pneumonia or hepatitis in combination with laboratory confirmation of Q fever (by serology or PCR). Strictly speaking, clinical pictures without fever (e.g. encephalitis) are not reportable but in practice, such cases are usually included as proven Q fever. Children were included in our study if they were reported by the MHS in Osiris and if they were younger than 15 years of age at disease onset. Osiris data do not contain clinical information, hospitalisation, or death. Therefore, one of us (AR), working at the MHS with the highest incidence of Q fever (MHS Hart voor Brabant), provided hospitalisation data on the cases reported to that MHS. For estimating incidence we used population data (2007–2009) from Statistics Netherlands (CBS).

### Estimated number of childhood infections

For an estimation of the number of new childhood infections during the period 2007–2009, we assumed equal exposure for children and adults [3, 5]. We used the adult seroprevalence increase (’07–’09) as an approximation for the seroprevalence increase in children. We used the 2006/2007 seroprevalence data from a random sample (5654 participants) of the Dutch population as the pre-epidemic control [15]. For post-epidemic adult data, we used the May 2010 Sanquin Blood Supply study. Sanquin tested over 40,000 serum samples of all consenting blood donors (18–65 years) living in the 23 postal code areas with the highest reported rates (23-HR-PC-areas) [16]. The Sanquin Blood Supply Foundation is the only organisation in the Netherlands authorised to manage the supply of blood and blood products. The 23-HR-PC-area is situated in the northeastern part of the province of Noord-Brabant, with 86,025 inhabitants of which 15,935 under the age of 15 years (<15 year: 15.6 %) in the epidemic years, i.e. 0.52 % of the total Dutch population of 16.4 million, and 0.54 % of all Dutch children <15 years of age (2.9 million).

We calculated the expected incidence in the high risk regions (23-HR-PC) of new childhood infections per 10,000 children (<15years) from the seroprevalence increase (from 2006 to 2010) and population data in the respective 23-HR-PC areas (2008) from Statistics Netherlands (CBS) [17].

### Estimated number of possible chronic childhood infections 2007–2009

The National Health Council of the Netherlands published a list of pre-existing conditions that carry a risk of developing chronic Q fever [18]. For children, only congenital heart- and/or vascular disease is listed as a relevant risk factor. In the Netherlands, data of children with congenital heart- and/or vascular disease are entered in the national congenital risk factors registry (CBS) [19]. Assuming an equal distribution of these conditions in the Netherlands, we estimated the potential number of children with congenital risk factors who could develop chronic Q fever after (un)detected infection in the 23-HR-PC areas.

### Comparison with literature: overview of case reports, seroprevalence studies and symptom rate

We reviewed the literature of the last 65 years (from January 1, 1949 (first published articles) to June 30, 2014) and used the PubMed/Medline and Scopus databases with the following combined text and MeSH heading search strategy: “Q fever” OR “*Coxiella burnetii*”, with a restriction for age {infant [birth-23 months), child, preschool (2–5), child (6–12), adolescent (13–18)]/all child (0–18)} and language [English, Dutch, French and German]. Different studies used different cut-off points for age, therefore a limit of 18 years and a selection of under 16 years was used to include as many studies of children as possible. References were scrutinized from these studies to identify other relevant studies (“snowball method”). We categorized articles according to the studied (sub-) populations i.e.: (A) general, (B) high exposure risk, and (C) symptomatic patients (i.e. with an influenza-like illness).

## Results

### Reported cases in the Netherlands during the epidemic years 2007–2009

In the period between 01/01/2007 and 01/01/2010, there were 3522 reported cases in The Netherlands, of which 44 were children aged 0–14 years (1.2 %).

**Fig. 1:**
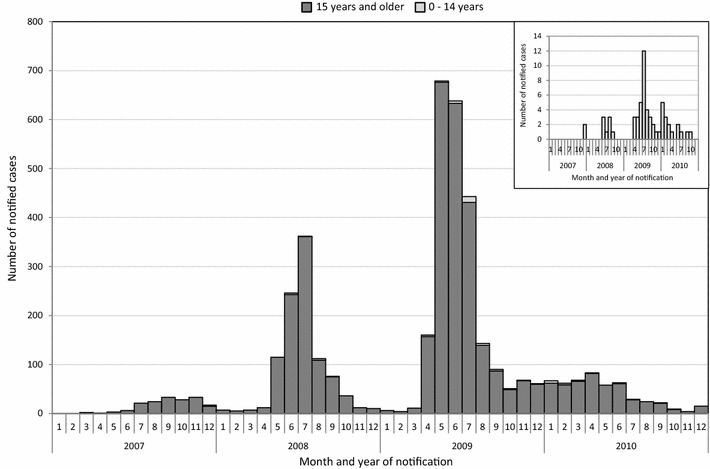
Number of notified cases during the years 2007–2010 for the ages of 0–14 years and 15 years and older

The 3-year Q fever reporting incidence for children aged 0–14 years was 0.15 per 10,000 inhabitants per year compared to 2.6 for adults (≥15 years). The number of cases increased per epidemic-year both in children and in adults; it showed a seasonal peak in late spring and early summer (Fig. 1a, b). More boys than girls were reported: 25 vs 19.

The geographical spread of childhood Q fever corresponded to that of adults [20]. From the 23-HR-PC-area, 22.7 % (10/44) children <15 years were reported, compared to 27.3 % (951/3478) adults (≥15 years).

The 44 reported children predominantly had fever with or without a headache. In 2007 and 2008, 11/23 (45.8 %) had pneumonia. Clinical data were not available for 2009. The MHS Hart voor Brabant reported 22 (of the total 44) children of whom four had pneumonia (18 %, as compared to 49 % in the adults); four children were referred to a local hospital for treatment (18 %, as compared to 24 % in the adults); one was admitted (4.5 %; adults 17 %) and fully recovered with appropriate treatment. No complications were reported.

### Estimated number of new infections in the epidemic years 2007–2009

The basic adult seroprevalence of Q fever antibodies in the pre-epidemic year 2006 was 2.4 % [21]. Post-epidemic adult donor seroprevalence in the 23-HR-PC-area was 12.2 % [16]. The increase in the three epidemic years was thus 9.8 % for adults, and we similarly used 9.8 % for children. In the 23-HR-PC-area 15,935 children under 15 years of age were living, suggesting that 1562 childhood infections (9.8 %) will have occurred in the three epidemic years. According to current literature [8, 9], roughly 15–50 % of infected children become symptomatic (including mild fever), i.e. an estimated 234–781 children may have shown symptoms in the 23-HR-PC area during the three-year period. However, from the 23-HR-PC area only 10 children were diagnosed and reported with Q fever in the three epidemic years.

### Estimated number of possible childhood chronic Q fever cases

Based on numbers of General Practices [22–24], the National Institute of Public Health and the Environment (RIVM, The Netherlands) estimated the 2007 absolute point prevalence of congenital heart- and/or vascular disease for the age group 0–14 years as 25,232 (0.004 % of all children 0–14 years) [25]. In the 23-HR-PC-area we thus expect there to be 137 children with a condition predisposing for chronic Q fever infection. If 9.8 % of these were indeed infected during the 3-year epidemic, we may expect 13 children with high-risk of developing chronic Q fever in the 23-HR-PC-area. So far, no children with chronic Q fever have been reported.

### Comparison with previous reports in literature

In the medical literature, Q fever in children is supposed to be asymptomatic in many cases. The most described clinical symptoms are an abrupt onset of fever accompanied with respiratory and/or gastro-intestinal symptoms. More severe manifestations are rare, but cases of hepatitis, hemolytic-uremic syndrome, myocarditis, pericarditis, cerebellitis, encephalitis, meningitis, hemophagocytosis, lymphadenitis, acalculous cholecystitis, osteomyelitis, vascular infection, skin disease and rhabdomyolysis have been described.

### Case studies, outbreaks and seroprevalence studies

Throughout the world, in 42 clinical reports, 140 cases of children with a serious outcome were described with one or more organs involved. In most cases, the lungs (n = 80), the heart (n = 24) or the bones (n = 23) were affected; four children reportedly died of their complications. In chronic Q fever, predominantly cardiac infections were described, less often neurological complications or osteomyelitis. Children have been mentioned in five outbreak reports, but no additional clinical data were given [26–30].

In 36 articles, data on Q fever seroprevalence in children were reported: 22 in A (the general childhood population), 4 in B (a high exposure risk) and 10 in C (symptomatic patients) (Additional files 1, 2, 3: Figures S1–S3 are annexed in the appendix).

The seroprevalence of antibodies against *C. burnetii* in children varied between 0 and 70 % in the different studies, but the data were hard to compare because of the differences in methods and cut-off’s used for testing. In general, the studies show an increase of seroprevalence with age.

African studies on childhood Q fever were initially rare, probably because Q fever is clinically difficult to distinguish from other feverish diseases [31–36]. The seroprevalence in Africa is highest in those countries where cattle farming is substantial [37].

In Japanese studies, children had a considerably higher seroprevalence compared to all other countries [38–41].

In Europe, a sero-epidemiological study among the local exposed population was performed in Switzerland after a huge outbreak [42]. All positively tested children were retrospectively interviewed about Q fever symptoms. Only 15 % of the 577 seropositive children under the age of 15 reported symptoms, compared to 64 % of the 2459 seropositive adults [7, 43].

### The Netherlands

Until now, only two epidemiologic seroprevalence investigations, described in five papers, have been performed in the Netherlands before [21, 44–47], three during [16, 48, 49] and two after [6, 50] the 2007–2009 epidemic.

In the first pre-outbreak study reflecting general exposure in the community, a cross-sectional seroprevalence study on sera from 1968, 1975, 1979 and 1983, the prevalence of antibodies in the age groups 1–4 (31 %) and 5–9 years (27 %) was comparable to that in adults (24 %) [44]. In this study, an indirect fluorescent immune-assay on IgM-antibodies against *C. burnetii* was used for the first time, which is a much more sensitive test than the previously used agglutination test on IgM-antibodies against *C. burnetii*.

In the later 2006 pre-outbreak study the seroprevalence was 2.4 % in a systematic random selection of the general Dutch population (PIENTER 2 [15, 21]). In this study, only 13 of the 1799 children under the age of 20 years showed serological markers of a previous infection (0.75 %).

In a pre-outbreak high risk seroprevalence study among inhabitants of dairy goat farms in 1984, 29 % of the children in the age group 0–14 years had IgG-antibodies against *C. burnetii* without a recognized course of disease, compared to 24.5 % in the adults (>15 years) [44]. In the third outbreak year, the seroprevalence in high risk participants at dairy goat farms was 68.7 %, and for children 12–17 years it was 57 % [48].

## Discussion

In order to inform and alert local paediatricians on the threat of silent chronic childhood Q fever we tried to estimate the expected number of these serious infections. An exposure survey or seroprevalence study might give the best answer to the question on how many chronic childhood infections can be expected. These methods are costly and a burden to the possibly exposed children. Therefore we first tried to estimate the expected number based on literature, exposure estimates, and high-risk estimates. We argue that childhood Q fever was probably seriously underdiagnosed in the Netherlands. In this overview of the Dutch Q fever epidemic (with 3522 reported cases in the 3-year epidemic period), we show that only 44 children (1.2 %) under the age of 15 years were reported. In this epidemic, Q fever did not present in the usual way as a disease of adult male farmers, veterinary surgeons, hide handlers, butchers or abattoir workers, but was transmitted equally to the inhabitants of the region surrounding dairy goat farms, with highest attack rates for those living nearest to the farms. As there were no childhood seroprevalence studies available, we used the adult data to estimate the number of acute and chronic childhood infections. The Netherlands is one of the most densely populated countries in the world. Airborne transmission from contaminated dairy goat farms to the neighbouring living areas was the greatest source in the Dutch outbreak. Therefore, inhabitants of all ages were: equally exposed, causing equal infection-incidences in men, women and children.

We estimated the number of symptomatic children in the high-risk area to be over two hundred possible cases (range 234–781), while in this area only 10 childhood cases were reported.

In medical literature, wide ranges of incident cases and seroprevalences exist, suggesting either true differences or differences in awareness or diagnostic possibilities. Wide ranges exist not only for children and adults per continent, but also within one country; e.g. for Australia the range is 1.3–2.5 % for children and 4–21 % for adults [51, 52]. This could reflect local exposure differences but might reflect differences in awareness and/or different lab techniques used with different cut off values. Example in the Richardus-study [44], using a sensitive indirect immune-assay on IgM-antibodies against *C. burnetii* [44] showed a seroprevalence of 15–65 %. While 20 years later in the 2006 study of Schimmer et al. [21] using IgG phase-2 antibodies against *C. burnetii* with an ELISA and corrected for confirmation with immunofluorescence, resulted in an estimated seroprevalence of 2.4 %. The high seroprevalence rates in the 80s are not corroborated with equally high numbers of Q fever. Therefore uncertainty remains about the cut-off values in the first study [44], that may have resulted in much higher seroprevalences in both adults and children as compared to all other studies.

In contrast to the world literature, serious complications were not described in any of the reported children in The Netherlands. In the Dutch reporting system, the patient’s condition is not reportable other than fulfilling the reporting criteria. Complications may have occurred, without being reported to the MHS, e.g. in children presenting without fever, as these do not fulfil reporting criteria. However, clinical presentations in the areas with highest risk showed no complications in any child [2]. In reported children, respiratory tract infections were predominant whereas in the medical literature children frequently only present with fever, headache or gastrointestinal symptoms (vomiting, abdominal pain, anorexia and/or diarrhoea) [46, 53, 54]. Also Wielders et al. found in adults that Q fever presented mainly with fever, cough and dyspnoea, in contrast to data from France and Southern Spain were hepatitis dominated [55]. Possibly, Dutch GP’s and paediatricians, following the testing guidance, predominantly tested children with respiratory problems. Another possible explanation might be a difference in *Coxiella* strain pathogenicity. Whether specific *Coxiella* strains cause different clinical symptoms is unknown [56–58]. In the Dutch outbreak, closely related MLVA genotypes A–H were found suggesting a clonal spread of *C. burnetii* [56–58]. The clinical importance of this finding remains unknown.

In the study of Porter [40], the authors mention as possible other explanation a greater sensitivity of Japanese children for *C*. *burnetii*. More important, the authors suggest that medical doctors are insufficiently aware of the broad variety in clinical presentations of Q fever in children and should test more often [40].

Contrary to acute Q fever, chronic Q fever is not notifiable in the Netherlands and at present there is no active surveillance system for chronic infections either. However, clinicians and medical microbiologists initiated a Dutch chronic database [59]. In this database, no childhood chronic Q fever was entered so far. Because chronic Q fever is a serious condition, it may be important to actively search for high-risk children in an early stage. In adults, the majority of proven and chronic Q fever during the Dutch epidemic was discovered in patients with a vascular infection or endocarditis [59]. Only 27 % of these patients showed signs or symptoms of a possible previous acute Q fever. Furthermore, screening of patients with a history of heart valve surgery for previous Q fever, showed signs of acute Q fever in 20 % and chronic Q fever in 8 % [60]. These data suggest that chronic Q fever might develop in asymptomatically infected children with additional risks factors such as congenital cardiac and/or vascular disease.

From current literature, it is unknown whether chronic Q fever will develop in as many children with a cardiac risk factor as in adults. Assuming similar risks in young and older adult cardiac patients, we expect that up to 13 children are at high risk for developing chronic Q fever in the area with highest risk (23-HR-PC). This high-risk area contributed a quarter of all reports, suggesting similarly a quarter of all exposures in the country. Subsequently we may expect over 30 children at high risk for chronic infections in other areas in the country. Therefore, we urge paediatricians in the exposed areas (especially the southern part of the Netherlands), but also across the boarders in Germany and Belgium where a similar *Coxiella* is found [57], to consider the possibility of chronic Q fever in these high risk children. Once found, it is important to report these cases to the MHS in order to broaden the data on the disease burden of this epidemic.

## Conclusion

The most common clinical presentation of Q fever in children is comparable to that in adults: a self-limiting disease with feverish symptoms. However, a high number of infections may have been asymptomatic and symptomatic cases might have gone undetected. As asymptomatic infections in high-risk children (cardio-vascular disorders) might develop into chronic Q fever, paediatricians should be aware of this. Vulnerable children can present, like adults, with exceptional and potential serious complications such as encephalitis, osteomyelitis, hepatitis or endocarditis. Although a rare disease, we recommend awareness of chronic Q fever in an early stage.

## Express information

Q fever in children is a rare disease for most paediatricians in the world, but according to local circumstances, paediatricians should be alert to it.

## Additional files

**Additional file 1: Figure S1.** Point estimates and confidence intervals, where available, for the seroprevalence in the general population.
**Additional file 2: Figure S2.** Seroprevalence in a high-risk population.
**Additional file 3: Figure S3.** Seroprevalence in symptomatic patient groups.

## Abbreviations

MHS: Municipal Health Services
NHG: the Dutch College of general practitioners
RIVM: the National Institute for Public Health and the Environment
PCR: polymerase chain reaction
CBS: Statistics Netherlands
23-HR-PC: the 23 postal code areas with the highest reported rates of Q fever
MLVA: multiple locus VNTR (variable-number tandem repeat) analysis
