# Supplementary figures and images for: Estimation of acute and chronic Q fever incidence in children during a three-year outbreak in the Netherlands and a comparison with international literature
Source: BMC Res Notes. 2015 Sep 18;8:456. doi: 10.1186/s13104-015-1389-0 (PMC4575485; doi:10.1186/s13104-015-1389-0)

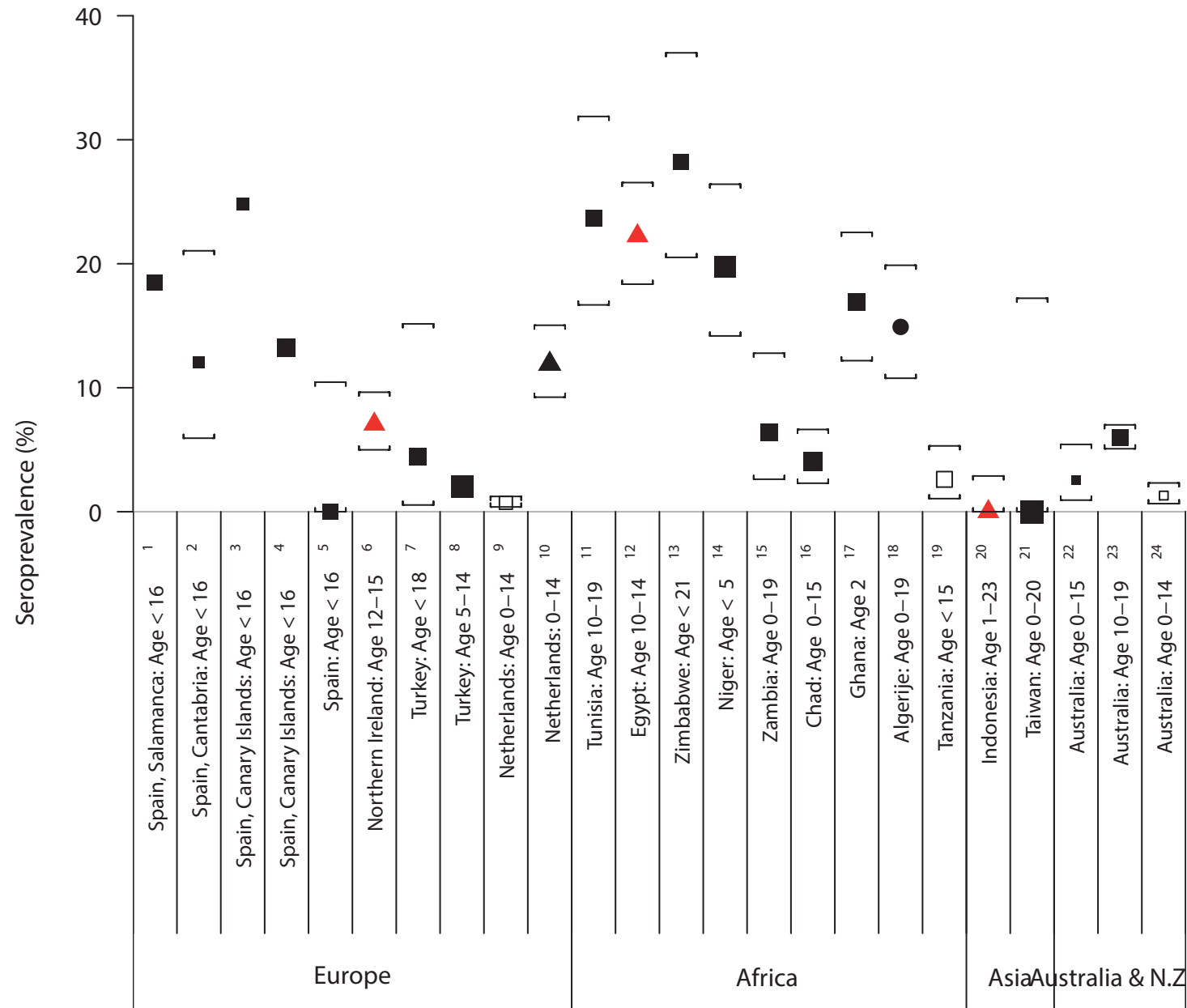

Supplement: Supplementary file 1 — Additional file 1: Figure S1. Point estimates and confidence intervals, where available, for the seroprevalence in the general population. [file 13104_2015_1389_MOESM1_ESM.pdf]

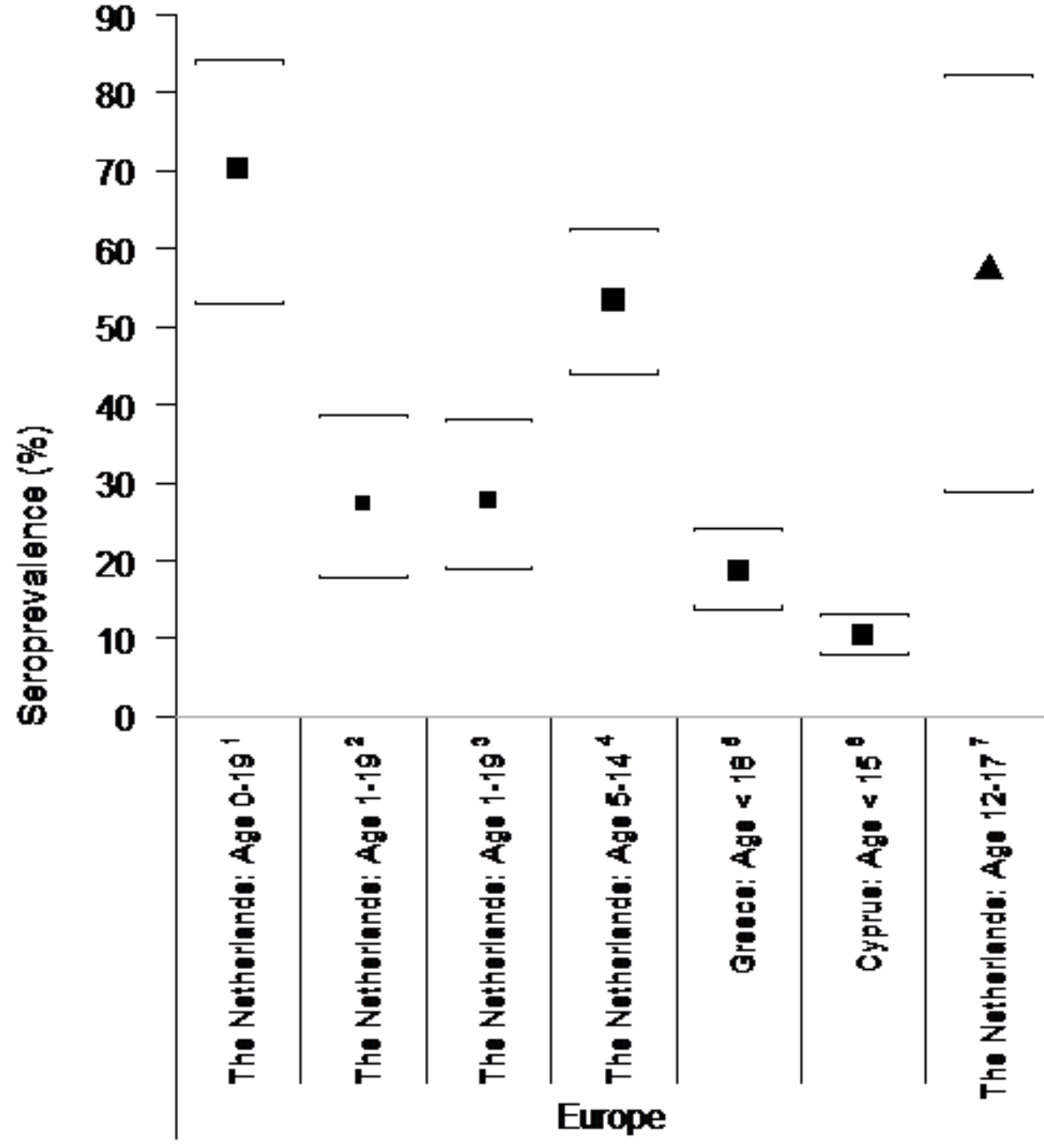

Supplement: Supplementary file 2 — Additional file 2: Figure S2. Seroprevalence in a high-risk population. [file 13104_2015_1389_MOESM2_ESM.pdf]

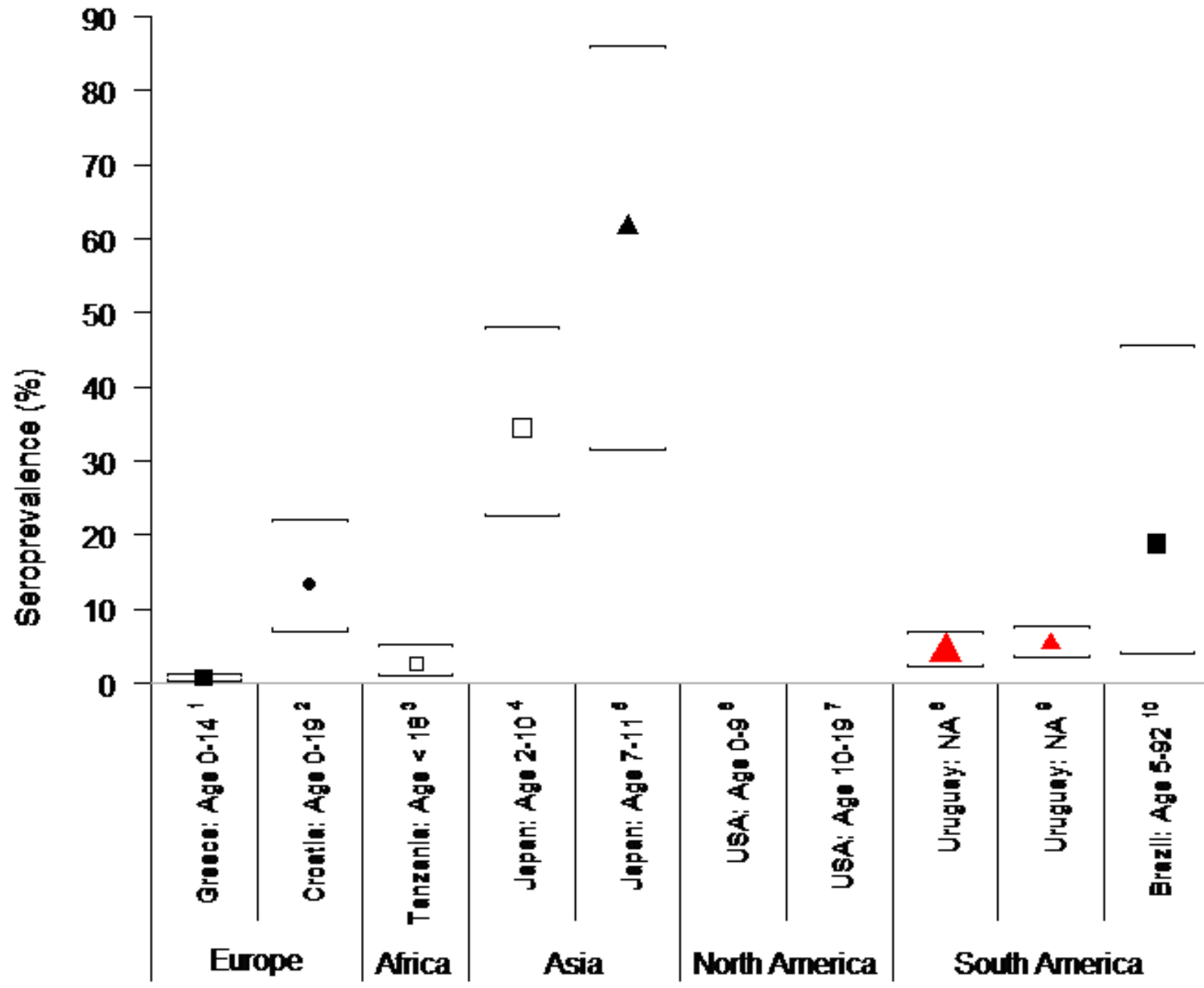

Supplement: Supplementary file 3 — Additional file 3: Figure S3. Seroprevalence in symptomatic patient groups. [file 13104_2015_1389_MOESM3_ESM.pdf]
